# Agriculture’s Contribution to Climate Change and Role in Mitigation Is Distinct From Predominantly Fossil CO_2_-Emitting Sectors

**DOI:** 10.3389/fsufs.2020.518039

**Published:** 2021-02-03

**Authors:** John Lynch, Michelle Cain, David Frame, Raymond Pierrehumbert

**Affiliations:** 1 Department of Physics, University of Oxford, Oxford, United Kingdom; 2 Centre for Environmental and Agricultural Informatics, Cran field University Cran field, United Kingdom; 3 New Zealand Climate Change Research Institute, Victoria University of Wellington, Wellington, New Zealand

**Keywords:** agriculture, climate change, climate policy, CO_2_, methane, nitrous oxide

## Abstract

Agriculture is a significant contributor to anthropogenic global warming, and reducing agricultural emissions—largely methane and nitrous oxide—could play a significant role in climate change mitigation. However, there are important differences between carbon dioxide (CO_2_), which is a stock pollutant, and methane (CH_4_), which is predominantly a flow pollutant. These dynamics mean that conventional reporting of aggregated CO_2_-equivalent emission rates is highly ambiguous and does not straightforwardly reflect historical or anticipated contributions to global temperature change. As a result, the roles and responsibilities of different sectors emitting different gases are similarly obscured by the common means of communicating emission reduction scenarios using CO_2_-equivalence. We argue for a shift in how we report agricultural greenhouse gas emissions and think about their mitigation to better reflect the distinct roles of different greenhouse gases. Policy-makers, stakeholders, and society at large should also be reminded that the role of agriculture in climate mitigation is a much broader topic than climate science alone can inform, including considerations of economic and technical feasibility, preferences for food supply and land-use, and notions of fairness and justice. A more nuanced perspective on the impacts of different emissions could aid these conversations.

## Introduction

The increased ambition of international climate policy, articulated in the Paris Agreement’s goal of “holding the increase in the global average temperature to well below 2°C above preindustrial levels and pursuing efforts to limit the temperature increase to 1.5°C above preindustrial levels” (UNFCCC, 2015), has increased scrutiny on the role all sectors can play in climate change mitigation. This has included a particular focus on agriculture (for example, in IPCC, in press). In addition, a number of recent high profile publications have highlighted agricultural emissions (e.g., Poore and Nemecek, 2018) and how they may need to be reduced to meet environmental commitments (e.g., Springmann et al., 2018). Yet in many treatments of agriculture’s role in climate change, some key principles appear to be increasingly overlooked or misunderstood: specifically, how the impacts of methane (CH_4_) and nitrous oxide (N_2_O), the major greenhouse gases emitted from agricultural production, are distinct from each other and, in particular, from carbon dioxide (CO_2_). An appreciation of these differences is important not only to understand what the mitigation of different gases can achieve in the context of the Paris temperature goal, but can also inform policy decisions. In this paper we outline the roles of these different greenhouses gases, consider how their reporting might be improved, and explore some of the potential implications for overall climate change mitigation.

## Agricultural Greenhouse Gas Emissions

Anthropogenic climate change is caused by multiple climate pollutants, with CO_2_, CH_4_, and N_2_O the three largest individual contributors to global warming (Myhre et al., 2013). Agriculture and food production is associated with all three of these gases, but direct agricultural emissions are unusual in being dominated by CH_4_ and N_2_O.

The global food system is responsible for ~21–37% of annual emissions (Mbow et al., in press), as commonly reported using the 100-year Global Warming Potential (more on this later). The composition of gases emitted by the food system does not reflect the overall global emissions balance, however, with agricultural activity generating around half of all anthropogenic methane emissions and around three-quarters of anthropogenic N_2_O (Mbow et al., in press).

Food system CO_2_ emissions are somewhat harder to quantify, due to the distinct processes through which they are generated and difficulty in applying uniform accounting methods or sectoral boundaries. A small amount of CO_2_ emissions occur directly from agricultural production, following the application of urea and lime, but these sources constitute an extremely small portion of total CO_2_ emissions. Energy-use CO_2_ from either agricultural operations (e.g., tractor fuel) or embedded in inputs (e.g., fertilizer manufacture and transport) can also be included as food system emissions, but are highly uncertain (Vermeulen et al., 2012), and are considered as energy or transport emissions within the IPCC (Intergovernmental Panel on Climate Change) accounting framework. The routes to reducing most of these emission sources are likely to be in the overall decarbonization of energy generation, rather than specific agricultural mitigations.

In addition, the food system is the main cause of ongoing land-use change CO_2_ emissions, primarily from clearing land for crop production or pasture. Net land-use related CO_2_ emissions are estimated as being responsible for around 14% of annual anthropogenic CO_2_ (Le Quéré et al., 2018), with 10% directly linked to agriculture (Mbow et al., in press).

A picture emerges of agriculture and the global food system as an important contributor to global greenhouse gas emissions: of CH_4_ and N_2_O in particular, but also significant amounts of CO_2_ depending on whether energy or land-use related emissions are included. Understanding the climate impacts of agriculture, particularly with respect to other sectors, necessitates understanding the distinct impacts of these three greenhouse gases.

## The Unique and Predominant Role of Carbon Dioxide Emissions in Anthropogenic Global Warming

Carbon dioxide is by far the main contributor to anthropogenic global warming (Myhre et al., 2013). This is not surprising given the enormous, and as of 2019 still increasing (Jackson et al., 2019), amount of CO_2_ that we emit every year. Yet it is not simply because emissions remain high that CO_2_ is responsible for so much warming. For every ton of CO_2_ we emit, a significant portion will remain in the atmosphere for millennia (Archer and Brovkin, 2008), and so the total amount of CO_2_ ever emitted by human activities commits us to a significantly altered climate essentially indefinitely, from any normal human decision making perspective (Clark et al., 2016). The extremely long-term persistence of CO_2_, and accumulating behavior that occurs as a result, is fundamental to our understanding of anthropogenic climate change, and is well-agreed upon by physical climate-carbon cycle models (Joos et al., 2013), but is not widely appreciated (Sterner et al., 2019).

This context reveals that achieving net-zero CO_2_ emissions is not simply a slogan to encourage ambitious emission reductions—it is a necessary condition of stopping global warming, stemming directly from our geophysical understanding of how contemporary CO_2_ emissions perturb the carbon cycle. This principle also suggests that in order to remain under a given temperature target there is a total, time-independent CO_2_ budget we must keep within (Frame et al., 2014). Such “cumulative carbon budgets” have increasingly provided an overarching framework for climate policy and a valuable tool to understand climate change (Rogelj et al., 2019). However, it also appears that there has been some confusion in how non-CO_2_ gases fit into this framework. As the cumulative carbon budget only applies to CO_2_, it follows that in addition to not exceeding the carbon budget, we must globally also limit the level of warming from all other sources to achieve the Paris Agreement. The IPCC’s Special Report on Global Warming of 1.5°C (IPCC, 2018) states that peak temperatures are dependent on cumulative CO_2_ emissions and non-CO_2_ radiative forcing, and suggests these non-CO_2_ contributions decline from their peak, but not do not have to reach net-zero emissions. We discuss next how shorter lived gases relate to global warming.

## Shorter-Lived Greenhouse Gases

The focus on reducing (to net zero) our CO_2_ emissions is well justified not just because it is the major anthropogenic climate forcer but also because it acts cumulatively. Shorter-lived greenhouse gases than carbon dioxide will, by definition, automatically be removed from the atmosphere over a shorter timeframe, so emissions will not continue to act cumulatively over the very long term that CO_2_ will. There follows two key implications for shorter-lived greenhouse gases in relation to CO_2_.

First, it suggests that shorter-lived GHGs have the potential for a sustained equilibrium concentration to be reached where constant ongoing emissions can eventually be matched by natural atmospheric removals.^1^ The timeframe at which this point is reached is determined by the atmospheric lifetime of the gas. For methane, such an equilibrium can be reached in decades, so we need to consider the gas as having a non-cumulative effect if we are to design a physically meaningful climate policy even in the near term, or simply to understand how past and present emissions affect the climate. The implication for a cumulative carbon budget is that pulse emissions of methane cannot be viewed as exhausting the budget in the same as way as pulse CO_2_ emissions. Rather, an ongoing rate of methane emissions will contribute to the budget in an equivalent manner to a pulse release of CO_2_ (Lauder et al., 2013; Pierrehumbert and Eshel, 2015; Allen et al., 2016; Cain et al., 2019; Collins et al., 2020).

For nitrous oxide it would take centuries to achieve this equilibrium between emissions and removals, so we would still need to treat emissions of the gas as acting approximately cumulatively in order to meet our climate policy targets over the next century. Over longer timeframes, such as the multicentennial timescales associated with ice sheet loss, we might want to consider ongoing nitrous oxide emissions as part of a long-term cycle, also distinct from the impacts of fossil fuel CO_2_.

In this context, “long-term” is relative. From the perspective of even the most far-sighted governments, multi-century climate policy seems fancifully long-termist, given that national infrastructure, economic, political, and emissions plans typically look not much further than 2050, by which point ambitions are already very vague. From a geological or Earth system perspective, however, a few centuries appears relatively brief compared to how long we anticipate it would take the Earth to recover from our CO_2_ emissions (Pierrehumbert, 2014; Clark et al., 2016). This brings us to the second key difference between CO_2_ and shorter-lived gases: the legacy of different emissions.

Even when net CO_2_ emissions are finally brought down to zero, we (humanity, including our descendants) will either be stuck with the climate impacts of these emissions for millennia, or face the burden of actively removing the enormous quantities of carbon that we have added. For shorter-lived gases, if we can stop emissions, then much of their impact will automatically be reversed over the timescales of their natural atmospheric removals. Thermal inertia in the climate response and the risk of hysteresis after crossing “tipping points” beyond which the Earth cannot readily return to its unperturbed state mean we cannot fully anticipate a complete reversal of impacts even from very short-lived gases. This is still distinct from the impacts of CO_2_, however, for which we not only have these long-term response elements, but also retain a portion of all past emissions in the atmosphere, continuing to exert a climate forcing.

## Co_2_-equivalent emissions

The principles outlined above are well-recognized in the climate science literature and physically uncontested. Misunderstandings or oversimplifications are not because of debate over these dynamics, but arise from our communication of different emissions as “CO_2_-equivalents.”

Non-CO_2_ gases are conventionally reported as CO_2_-equivalent emissions (“CO_2_-e”) using the 100-year Global Warming Potential (GWP100). This metric is based on the total perturbation to the atmospheric energy balance (radiative forcing) by an idealized pulse-emission of different gases over the 100-years following this pulse, scaled relative to CO_2_ (Myhre et al., 2013). The limitations of this metric have been discussed in detail elsewhere (for recent examples, see Pierrehumbert, 2014; Allen et al., 2016; Tanaka and O’Neill, 2018; Wigley, 2018). Here we simply emphasize some particularly fundamental points building on the observations above. First, by describing all emissions as direct equivalents using single, static weighting factors, conventional application of GWP100 (or any other pulse-based metric taking this approach), misses dynamics that are driven by changes in the rate of emissions, and in particular cannot distinguish the cumulative and non-cumulative nature of different gases. Second, even for what we can infer from the impacts of isolated pulse-emissions, GWP is blind to any impacts beyond its stated timeframe, and so does not reveal the differing legacies of emissions—including the contemporary legacy of past emissions.

**Figure 1:**
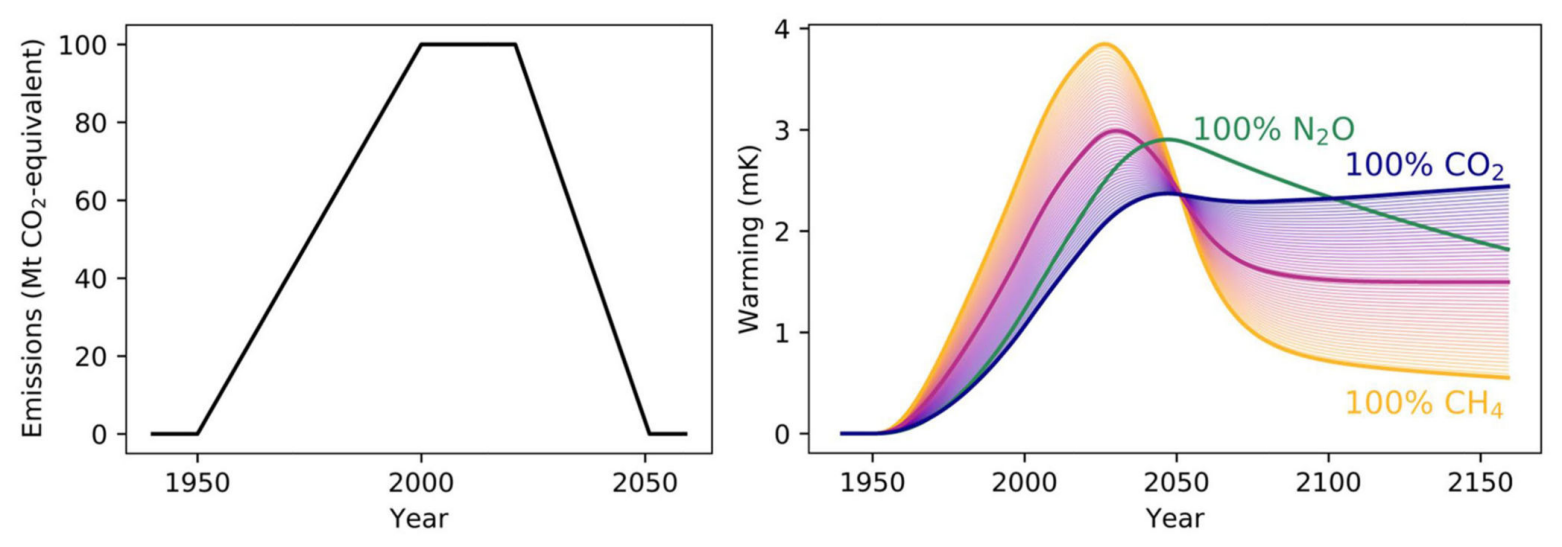
A single emissions pathway (left) reported as CO_2_-equivalents using the 100-year Global Warming Potential (GWP100) can have very different impacts (right) depending on the gas-specific composition, illustrated by showing the warming contribution if the CO_2_-equivalent emissions are entirely nitrous oxide (green), entirely carbon dioxide (blue), entirely methane (orange), or various combinations of carbon dioxide and methane (blue-to-orange spectrum; 50% methane, 50% CO_2_ shown as stronger purple line).

Figure 1 illustrates some of these points but also draws attention to perhaps an even more important consideration: the extremely ambiguous warming impacts of emissions reported using the GWP100. This figure was generated using the FAIR simple climate model (Smith et al., 2018) in its default set-up, adding the stated CO_2_-equivalent emissions as either nitrous oxide, methane, or CO_2_ (or balances thereof) to RCP4.5 emissions, then deducting the modeled warming from the baseline RCP4.5 conditions to show the impacts of these emissions alone. GWP100 values of 265 and 32 were used for nitrous oxide (Myhre et al., 2013) and methane (Etminan et al., 2016), respectively.

It is immediately clear that emissions scenarios reported as CO_2_-equivalents do not indicate an unambiguous warming path. Common statements such as “methane is an *x* times stronger greenhouse gas than CO_2_” are inherently oversimplifying, as they cannot capture the contrasting dynamics of the two gases. Regardless of whether one might argue GWP100 CO_2_-equivalent emissions still have a use in climate policy or as simplifying communication tool, it undeniably fails as a universal environmental indicator, shown by the very large spread of possible temperature responses to supposedly equivalent emissions. We should not use such an imprecise measure in scientific contexts, but this is more often than not how emissions are reported: researchers routinely discard essential climatic data by not reporting individual gases separately (Lynch, 2019).

The emissions pathway here—increasing over the second half of the twentieth century, stabilizing briefly and then rapidly falling to zero emissions by 2050—can be thought of as a providing an illustration of the warming that has resulted from anthropogenic emissions and their roles in ambitious mitigation (in terms of overall profile; it is not representative of the scale of different emissions). Exploring what the figure shows can therefore be informative as to the role of different gases, and highlight what we would get wrong by considering all emissions as directly analogous to CO_2_.

The rate at which emissions initially increase results in methane having a much greater impact than the nominally equivalent amount of CO_2_ would indicate. In an agricultural context, such rapid increases occurred for ruminant methane emissions over the past century, and their reported CO_2_e likely underestimates their contribution to current warming (Reisinger and Clark, 2018). In general, the impact of increasing methane emissions at rates above ~1% per year will be understated by reporting using GWP100 CO_2_e (Lynch et al., 2020a).

As emissions start to decline from 2020 to 2050, and then stay fixed at zero, an even starker difference between the gases becomes clear. As methane emissions are reduced, most of the warming they caused is reversed. The short lifetime of the gas means that the concentration of methane in the atmosphere falls when not maintained by ongoing emissions. Meanwhile, for CO_2_, stopping emissions ends the ongoing temperature increases that result from any non-zero emissions, and we end with a relatively fixed level of long-term warming.

Reducing CO_2_ emissions to zero is therefore necessary to prevent further warming, but for methane, completely eliminating emissions goes beyond what is required for temperature stabilization. A “net-zero” CO_2_ emitter will continue to exert a significant climate impact long after their emissions cease, potentially much greater than a methane emitter who can only manage a partial emission reduction. So if we reach zero emissions of the two gases, a methane emitter has contributed a much greater role in climate change mitigation than a nominally “equivalent” CO_2_ emitter, and this continues to be the case into the very long-term.

An alternative perspective on these dynamics can be gained by considering why they are not captured by the GWP100. As it covers a period of 100-years, the GWP100 is effectively open-ended for CO_2_, but not for methane: for CO_2_ there is relatively consistent warming contribution across the 100-year period after emission and well beyond, but for methane the impacts of an emission are largely experienced within the first few decades. As it is integrates total forcing over the 100-year period to a single value, the GWP100 undervalues the initial impact of a methane emission, but then also fails to clearly reflect that most of this initial impact is then reversed. To capture the difference between CO_2_ and methane emissions with this dynamic detail, then, we could instead consider an individual methane emission as being equivalent to a large CO_2_ release, but with a large CO_2_ removal occurring shortly afterwards (Lynch et al., 2020a). To have a truly equivalent effect to a methane emitter reducing their emissions, a CO_2_ emitter would therefore not only need to reduce their emission rates but also actively recapture most of their past emissions.

The overall temperature change contribution and eventual warming legacy of different actors (be it nations, sectors, or individuals emitting different combinations of GHGs) thus cannot be inferred from emissions in a given year or whether or not they have an eventual “[net]-zero” ambition, as climate is shaped (in a gas-specific manner) by all past emissions. Yet annual emissions and net-zero targets have become the common currency of climate change communications and policy discussions.

Clearly it is still climatically beneficial to reduce methane emissions as much as we can, provided this is not at the expense of stopping CO_2_ emissions. However, the question of how much methane emissions must or should be expected to reduce by, especially in relation to what CO_2_ emitters have now achieved by stopping emissions, is revealed as less physically straightforward than might be assumed if all gases really were directly equivalent.

For N_2_O, the dynamics are approximately intermediate to those of CO_2_ and methane. The initial impact of increasing emissions is undervalued if comparing to a nominally equivalent amount of CO_2_, and in the longer-term the automatic reversibility of warming from N_2_O is also not reflected. The reader can imagine a similar spread of possible warming between 100% N_2_O and either CO_2_ or CH_4_ to again emphasize the ambiguity emerging from using GWP100 CO_2_e to report emissions. Over this two-century example, the behavior of N_2_O is closer to that of CO_2_, however, and so, as noted above, N_2_O can be treated as a cumulative pollutant in short/medium-term climate policy without giving a misleading indication of its impacts, unlike methane.

## Communicating Emissions

The significant limitations of reporting only GWP100 CO_2_e lead us to suggest changes in how to communicate emissions and related concepts. The phrase “carbon emissions” is often used to refer either to carbon dioxide emissions or as shorthand for “all greenhouse gas emissions” (this second usage likely arising from either the dominance of CO_2_ as a contributor to global warming, or the ubiquitous usages of “CO_2_ equivalents”). This ambiguity in meaning has perhaps led to or cemented some misconceptions around the direct fungibility of different gases, but could easily be overcome by using “carbon emissions” to refer exclusively to carbon dioxide, while using the more precise “greenhouse gas emissions” (or often simply “emissions,” depending on the context) when discussing non-CO_2_ emissions or combinations of multiple gases.

Clear and appropriate terminology is even more important in the context of “carbon budgets.” In the climate science literature, cumulative carbon budgets are CO_2_-only, as they result from the cumulative nature of CO_2_ emissions outlined above, and particularly the near-linear relationship observed between cumulative CO_2_ emissions and their contribution to global warming (Matthews et al., 2018). Confusingly, in the policy context, “carbon budgets” are instead usually used to denote aggregated GWP100 CO_2_-equivalent ambitions, as in the UK government’s “carbon budgets,” which define reductions in all greenhouse gases over time. Increased clarity is required, particularly from researchers, to avoid these misinterpretable terms. In a scientific context, “carbon budgets” should be used exclusively for CO_2_, or when using alternative equivalence approaches such as GWP* CO_2_-warming equivalents (Cain et al., 2019), CO_2_-forcing equivalents (Jenkins et al., 2018), or CGWP/CGTP (Collins et al., 2020) that can report short-lived gases in a way that is compatible with cumulative carbon budgets.

These concerns are particularly notable in light of recent focus on “carbon neutral” and “[net-]zero carbon.” As explained above, the need for net-zero emissions in order to stabilize global temperatures is CO_2_-specific and comes directly from our understanding of how cumulative CO_2_ emissions affect the climate. It can become unclear what is inferred by “carbon neutrality” (or similar terms), as it has different implications for non-CO_2_ gases depending on whether it refers to temperature stabilization (the objective and outcome of becoming “CO_2_ neutral”), or net-zero emissions (the CO_2_-specific requirement for temperature stabilization).

## Role of Agricultural Emission Reductions in Climate Change Mitigation

### Global Emission Reductions

Decreasing agricultural greenhouse gas emissions is important— net food system CO_2_ emissions must be eliminated, as with all other CO_2_ emissions, and reducing agricultural methane and N_2_O, while distinct from CO_2_, is climatically beneficial and must be encouraged. Atmospheric concentrations of both methane (Nisbet et al., 2019) and N_2_O (Tian et al., 2020) resemble their “worst-case” representative concentration pathways (RCPs). To achieve the climate objectives of the Paris Agreement, all sectors must make large-scale, rapid efforts to decrease their emissions of all gases (Rogelj et al., in press). Insufficient agricultural emission reductions will compromise our ability to limit global warming to 1.5 (Leahy et al., 2020), and current trajectories for food system emissions threaten this target by themselves (Clark et al., 2020).

Despite this context, there remain many questions over exactly how targets should be set for different greenhouse gases. At the level of global emission reduction requirements, it has been suggested that, though not explicitly stated, the Paris Agreement should be interpreted in terms of achieving net-zero greenhouse gas emissions aggregated using the GWP100 (Schleussner et al., 2019). Others have argued that there are multiple interpretations of how different gases should be balanced (Fuglestvedt et al., 2018), or that the Agreement should be refined with a more specific focus on net-zero CO_2_, given that net-zero emissions across all gases is not a physical requirement for the Agreement’s temperature targets (Tanaka and O’Neill, 2018). These points can be contested as, for the reasons illustrated above, targets based on the GWP100 do not have a clear link to temperature outcomes. There are risks in taking an approach based on policy accounting tools rather than the temperature goal itself.

As different gases are not truly “equivalent” to one another, substituting action to reduce emissions of one gas with greater efforts on another does not result in the same outcome. It has been highlighted that reducing methane emissions at the expense of CO_2_ is a short-sighted approach that trades a near-term climate benefit with warmer temperatures for every year thereafter (Pierrehumbert, 2014), and reducing methane emissions only limits peak warming when we are at or approaching net-zero CO_2_ emissions (Bowerman et al., 2013). A GWP100 accounting based framework does not reveal these temporal details (Lynch et al., 2020a). In an agricultural context there are risks we might trade shorter-for longer-lived gases by supporting certain products or types of production over others, but an even greater danger is that action taken on agricultural emissions might reduce the focus on decarbonization. If strong efforts are made to reduce agricultural emissions but prove expensive—in terms of monetary costs, political capital, public goodwill, or individual effort—and detract from efforts to eliminate fossil CO_2_ emissions then we will be climatically worse-off.

### Sectoral Roles

Even if we did have universally agreed global emission requirements, there remain political questions regarding how this should be achieved across different sectors (i.e., agriculture vs. energy) and nations, and we suggest the distinct physical impacts of different gases should be kept in mind when allocating emission reduction commitments. So, for example, while reducing methane emissions lowers temperatures by undoing previous contributions to warming, fully removing all methane emissions is not a physical requirement to prevent any further increases in temperature, as it is for CO_2_. The extent to which we do *need* to limit agricultural methane emissions below current levels to keep warming under 1.5°C is therefore not because they alone will, if sustained at current rates, exceed this threshold. Rather, we need to reduce agricultural methane emissions because they are still increasing (FAO, 2019), and we do not anticipate sufficiently rapid decarbonization that simply limiting non-CO_2_ warming to current levels will be sufficient. We must likely also reverse some extant warming from agricultural methane and actively remove CO_2_ from the atmosphere to meet our climate commitments (Rogelj et al., in press).

The appropriate balance of these actions—stopping and/or reversing warming from methane or CO_2_-is not a question that physical science can resolve. For example, how much should consumption of ruminant products be reduced in order to lower methane emissions and permit extra CO_2_ before net-zero emissions can be reached?^2^ There are many emission pathways resulting in the same eventual climate outcomes. Very rapid energy decarbonization could negate the need to significantly reduce ruminant methane emissions below current levels, yet still meet an ambitious temperature target. Alternatively, dramatically cutting ruminant methane emissions could reverse significant amounts of present-day warming, allowing a substantial amount of required or more cost-effective CO_2_ emitting activities to occur before exceeding the same temperature threshold. The optimal strategy depends on when and at what scale alternative energy generating technologies are available, the economic value of these ruminant emissions compared to CO_2_ generating activities, and simply how socially and politically acceptable it will be to limit one activity compared to the other. Parties to the Paris Agreement “recogniz[e] the fundamental priority of safeguarding food security and ending hunger, and the particular vulnerabilities of food production systems to the adverse impacts of climate change” (UNFCCC, 2015). Any robust mitigation strategy, whether model-based or negotiated, should ensure that sufficient agricultural production remains (and hence generates emissions) to feed the human population, but beyond that obvious requirement, trade-offs may appear, and need to be set out. Changing dietary behaviors, particularly reducing the consumption of animal products, should result in significant emission mitigations, alongside wider environmental and health benefits (Mbow et al., in press). Removing ruminant emissions would increase the CO_2_ emission budget for a given temperature target, and so could delay the speed at which a global shift to renewable energy must occur, reducing the cost of this transition; but may also entail negative impacts on, for example, consumer welfare and farmer incomes (Bryngelsson et al., 2017). Mitigation beyond the level at which co-benefits are experienced needs to be considered in a rounded, informed, transparent fashion, especially where there is the potential for temporal climate trade-offs to arise (e.g., mitigation of methane leading to greater emissions of either nitrous oxide or carbon dioxide).

### Integrated Assessment

Emission reduction pathways intended to answer the questions posed above are primarily generated and/or assessed using climate-economic integrated assessment models (IAMs), but these have been heavily criticized for their opacity (Robertson, 2020). It also been argued that mitigation assessments have emphasized technological and economic feasibility but done little to address behavioral, cultural, or social plausibility, with dietary choices noted as a key example (Nielsen et al., 2020). We are currently failing to implement the policy tools that modeled pathways use to bring down agricultural emissions (Leahy et al., 2020). We must do more do explore what is preventing the implementation of agricultural emission reductions and consider how this problem is best overcome: stronger agricultural interventions or redoubled effort to speed emission reductions in other sectors, where we have no choice but to eventually eliminate emissions regardless of efforts made elsewhere (recognizing that to keep to the most stringent climate targets both of these approaches must be rapidly escalated).

In this context, we note that the recent focus has been on the role of agriculture in emission scenarios that keep warming to within 1.5 or 2° C warming above pre-industrial temperatures (Roe et al., 2019). We should strive for the largest mitigation effort we can, but these are extremely ambitious mitigation targets, and not all integrated models even suggest it is possible to reach them. Meeting these targets is dependent on the complete decarbonization of energy generation occurring imminently, but until 2019 CO_2_ emissions were still increasing (Jackson et al., 2019), and 2020 is only anticipated to show a small decline as a result of the large-scale disruption wrought by COVID-19 (Le Quéré et al., 2020). Furthermore, this decline is likely to be temporary, yet we will need continued year-on-year CO_2_ emission reductions of a similar magnitude to remain under 1.5 degrees (Le Quéré et al., 2020). Achieving the stringent agricultural mitigations proposed in ambitious scenarios mitigation pathways is no guarantee of meeting, or even coming close to, these temperature targets. Should we miss these goals, we must reset our expectations and consider what is now politically and practically workable across different sectors to salvage the maximum mitigation effort, making the concerns identified above even more important. If we are committed to a GWP100 accounting based approach above all else—a highly prescriptive yet physically abstract approach to setting emission reduction targets—we may lose flexibility in changing tack.

We contend that the role of different emissions, and by extension different sectors, in mitigating climate change should be driven by and understood in terms of their temperature outcomes. Success should not be measured via an abstract and highly ambiguous reporting unit, whose primary virtue is customary use. Simplified means of communicating emissions or emissions targets often obscure their climate impacts and omit the wider considerations that might be important for informed decision making. Similarly, historic and anticipated warming from different actors is important to address many concerns over equitable climate policy, as has been highlighted in discussions of equity and responsibility of different nations to mitigate climate change (Matthews et al., 2014), but not featured particularly clearly regarding different activities. The discourse over the roles and responsibilities of different sectors currently revolves around proportions of annual emissions aggregated using the GWP100 and when “net-zero” emissions might be achievable. We argue that the exploring the sectoral and national attribution of overall warming to date and across alternative scenarios is a more intuitive and politically salient measure.

Finally, we must also briefly note the importance of wider land-use considerations linked with agricultural emission reductions. While a full treatment of this topic is beyond the scope of this paper, land-use for climatic benefits such as carbon sequestration or biomass for energy is often highlighted as being critical for ambitious mitigation pathways (IPCC, in press). Recognizing that agricultural land is not being used primarily for these purposes, a “carbon opportunity cost” is increasingly cited for agricultural production (Searchinger et al., 2018). Interventions to reduce agricultural emissions may therefore also be linked to land-use based mitigation efforts (or vice-versa). Greater attention must be paid to the drivers and implications of alternative land-uses, as it is through different land managements that agricultural emission reduction strategies can support or conflict with other Sustainable Development Goals (Arneth et al., in press). This further highlights some of the difficulties but also the importance of clear and robust discussion over what agricultural transitions are feasible and desirable. There are many inter-related concerns around agriculture, and particularly livestock (Lynch et al., 2020b), but we reiterate that a more direct link between policy interventions and climate outcomes would be helpful for these conversations.

## Conclusions

The non-CO_2_ gases methane and nitrous oxide comprise a uniquely large share of agricultural emissions. We therefore need to appreciate how emissions of these gases contribute to temperature change in order to understand the role of agriculture in global warming, and what agricultural emission reductions can achieve. There is no satisfactory means by which a single pulse-emissions-based weighting can be used to describe a physical “equivalence” between gases, so our common reporting measure of GWP100 CO_2_e, which is built on this approach, cannot provide clear climatic inference. These limitations are well-recognized: Fuglestvedt et al. (2000) noted “it is uncertain whether policy makers are aware of the significance of lifetime differences and the shortcomings associated with the GWP methodology.” We highlight these same concerns for environmental and food sustainability research, where in many cases emissions metrics are used in ways which are at best ambiguous and at worst positively erroneous. More attention should be paid to the uses and limitations of different metrics for different purposes. We call for more environmentally robust approaches in the future, including the use of multiple and alternative emission metric approaches, and modeling of the relevant impacts.

Revisiting the reporting of emissions, and appreciating that agricultural emissions are not direct analogs of fossil CO_2_, might also encourage a more critical take on some of the approaches and assumptions that agricultural mitigation requirements are built upon. Climate science tells us what different mitigation options *can* achieve-it does not directly inform on what mitigations *must* be made, except for the principle, which emerges directly from geophysics, that CO_2_ emissions *must* eventually reach net-zero to prevent further warming. There may be political discussions on how quickly net-zero CO_2_ emissions can be reached, or how the limited cumulative emissions budget can be equitably shared out, but there is a clear ultimate requirement. For agricultural methane, and to some degree nitrous oxide, there is scope to negotiate what ongoing “sustainable” emission rates might be acceptable for different actors. Clarifying the impacts of different emitters can facilitate these negotiations and lead to workable mitigation policies. Other elements that need to be considered in balancing emission reductions from different sectors require broader political, ethical, and social considerations, and we encourage researchers in these areas to be open and transparent about these factors.

## Footnotes

1 We note here that the primary methane destruction process is oxidation to CO_2_, but for biogenic methane, such as agricultural emissions, this returns atmospheric CO_2_ that was recently fixed as plant biomass via photosynthesis. This is in contrast to the oxidation of fossil methane (“natural gas”), which does represent an additional, but small, CO_2_ source. This distinction was recognized in the IPCC 5th Assessment Report, resulting in different Global Warming Potentials of biogenic and fossil methane (Myhre et al., 2013).

2 The same argument could be made for substituting rice for other cereals without a significant methane footprint, but ruminant livestock are responsible for a larger share of anthropogenic methane emissions, and most research and advocacy on reducing dietary methane emissions focuses on ruminants.
